# Effective therapeutic regimens in two South Asian countries with high resistance to major *Helicobacter pylori* antibiotics

**DOI:** 10.1186/s13756-019-0482-x

**Published:** 2019-02-15

**Authors:** Muhammad Miftahussurur, Hafeza Aftab, Pradeep Krishna Shrestha, Rabi Prakash Sharma, Phawinee Subsomwong, Langgeng Agung Waskito, Dalla Doohan, Kartika Afrida Fauzia, Yoshio Yamaoka

**Affiliations:** 1 Division of Gastroentero-Hepatology, Department of Internal Medicine, Faculty of Medicine-Dr. Soetomo Teaching Hospital, Universitas Airlangga, Surabaya, 60131 Indonesia; 2 Institute of Tropical Disease, Universitas Airlangga, Surabaya, 60115 Indonesia; 3 Department of Gastroenterology, Dhaka Medical College and Hospital, Dhaka, Bangladesh; 4 Department of Gastroenterology, Maharajgunj Medical Campus, Tribhuvan University Teaching Hospital, Kathmandu, 44600 Nepal; 5 Department of Environmental and Preventive Medicine, Oita University Faculty of Medicine, 1-1 Idaigaoka, Hasama-machi, Yufu-City, Oita 879-5593 Japan; 6 Global Oita Medical Advanced Research Center for Health, Oita University, Oita, 870-1192 Japan; 7 Department of Medicine, Gastroenterology and Hepatology Section, Baylor College of Medicine, Houston, TX 77030 USA

**Keywords:** Nepal, Bangladesh, Drug resistance, *Helicobacter pylori*, Antibiotics

## Abstract

**Background:**

Nepal and Bangladesh have a high prevalence of *Helicobacter pylori* with high resistance rates to clarithromycin, metronidazole, and levofloxacin. Here, we evaluated the susceptibility and genetic mutations of 5 alternative antibiotics against isolates from both countries to obtain an effective treatment regimen for *H. pylori* eradication.

**Methods:**

We used the agar dilution method to determine the minimal inhibitory concentration of 5 alternative antibiotics against 42 strains from Nepal and 56 from Bangladesh and performed whole genome mutation analysis.

**Results:**

No resistance to furazolidone or rifabutin and a high susceptibility of sitafloxacin (95.2% in Nepal and 98.2% in Bangladesh) were observed. In contrast, resistance to rifaximin (52.4% in Nepal and 64.3% in Bangladesh) was high. Moreover, resistance to garenoxacin was higher in Bangladesh (51.6%) than in Nepal (28.6%, *P* = 0.041), most likely due to its correlation with levofloxacin resistance (*P* = 0.03). Garenoxacin and rifaximin were significantly correlated in Bangladesh (*P* = 0.014) and occurred together with all sitafloxacin-resistant strains. Mutations of *gyrA* could play a significant role in garenoxacin resistance, and double mutations of A87 and D91 were associated with sitafloxacin resistance. Analysis of the *rpoB* gene demonstrated well-known mutations, such as V657I, and several novel mutations, including I2619V, V2592 L, T2537A, and F2538 L.

**Conclusions:**

Rifabutin can be cautiously implemented as therapy for *H. pylori* infection due to its interaction with the tuberculosis endemic in Bangladesh. The high susceptibility of furazolidone and sitafloxacin suggests their possible future application in Nepal and Bangladesh.

**Electronic supplementary material:**

The online version of this article (10.1186/s13756-019-0482-x) contains supplementary material, which is available to authorized users.

## Background

*Helicobacter pylori* chronically affects half of the worldwide population and remains a significant global problem due to its role in the pathogenesis of peptic ulcer diseases and gastric cancer [1]. South Asia is the most densely populated region in the world, with a total of 1,891,454,121 residents in 2017 (http://www.worldometers.info). Several countries in this region have a high prevalence of *H. pylori.* The prevalence in Bangladesh was 60.2% with a high re-infection rate [2, 3] and 73.4% in Bhutan [4]. The high prevalence of *H. pylori* in this region was associated with gastroduodenal diseases, especially peptic ulcer diseases; thus, the cost of eradication therapy is high. A strategy for achieving successful therapy is needed not only to eradicate *H. pylori* infection but also to improve clinical symptoms in gastroduodenal diseases and halt their subsequent progression to gastric cancer [5, 6]. However, this goal has been significantly challenged by increased rates of antibiotic resistance to clarithromycin, metronidazole, and levofloxacin in South Asian countries, primarily in first- and second-line antibiotics to combat *H. pylori* infection [7]. Even though *H. pylori* treatment was previously administered to patients with clinical manifestations, the recent Maastricht V Consensus recommends treatment for all positively infected patients [8]. Therefore, an effective regimen needs to be established because the inappropriate use of antibiotics triggers resistance to other microorganisms.

We reported high resistance to clarithromycin and metronidazole (39.3 and 94.6%, respectively) in Bangladesh but low resistance to amoxicillin and tetracycline [7]. In addition, high resistance to metronidazole and clarithromycin (88.1 and 21.4%, respectively) is present in Nepalese strains [9]. Increasing resistance to levofloxacin (66.1% in Bangladesh), which is still used in second-line regimens and as rescue treatment for *H. pylori* eradication in South Asia, was also observed. Importantly, the resistance rate to antibiotics in both countries exceeded the threshold of high resistance rates defined by the Maastricht V Consensus guidelines on the management of *H. pylori* infection (15% for clarithromycin and 40% for metronidazole) [8], with high resistance to levofloxacin suggesting that clarithromycin- or metronidazole-based regimens and a levofloxacin-based regimen are insufficient as first- and second-line eradication therapy for *H. pylori,* respectively. We attempted to determine why the *H. pylori* cure rate in South Asia is approximately 70% [10], which is lower than the recommended eradication target (90–95%) [11].

Furazolidone is considered an alternative drug [8] due to its efficacy, low rate of primary bacterial resistance, and low cost [12]. Rifabutin, an anti-tuberculous agent inhibiting transcription of *H. pylori* [13–15], may become an alternative treatment regimen [16]. Few data exist on rifaximin, a derivative of rifamycin and also rifabutin; however, it is a promising anti-*H. pylori* drug due to its poor absorption in the blood with minimal adverse effects and high bioavailability in the gastrointestinal tract [8, 17]. Garenoxacin, a des-fluoro [6] quinolone [18], and sitafloxacin, a more potent third-generation quinolone, were also reported to be effective for *H. pylori* [19, 20].

Bangladesh and Nepal are 2 significant countries that could be used as a population model for high resistance to clarithromycin and metronidazole. Resistance data from both countries would provide information regarding proposed alternative regimens worldwide. Furthermore, investigating alternative treatment methods is necessary in order to determine the best regimen. Evaluating antibiotic susceptibility tests and performing molecular analysis is important in understanding the pattern and mechanism of resistance [21]. We provided data regarding optional antibiotics with low or uncommon resistance rates and analyzed DNA sequences to identify mutation candidates playing a role in antibiotic resistance.

## Methods

### Patients and *H. pylori*

We used the same *H. pylori* strains from our previous studies [7, 9, 22]. Briefly, we included Nepalese and Bangladeshi adult dyspeptic patients undergoing endoscopy in the Gastroenterology Department at Tribhuvan University Teaching Hospital (TUTH), Kathmandu, Nepal, and Dhaka Medical College, Dhaka, Bangladesh [7, 9, 22]. Subjects with any history of gastric resection, those taking *H. pylori* eradication therapy*,* and those receiving treatment with proton pump inhibitors, H2-receptor blockers, or bismuth-containing compounds at 4 weeks before the examination were excluded from the study, and gastric biopsy specimens were collected. Overall, 42 strains (146 subjects) from Nepal (35 gastritis, 4 duodenal ulcers, and 3 gastric cancer) and 56 strains (133 subjects) from Bangladesh (53 gastritis and 3 peptic ulcers) were successfully isolated from homogenized antral biopsy specimens by inoculating into a selective agar plate and incubated up to 10 days under microaerophilic conditions (10% O_2_, 5% CO_2_, and 85% N_2_) at 37 °C. The growing *H. pylori* was subsequently subcultured into antibiotic-free Mueller–Hinton II agar medium (Becton Dickinson, Franklin Lakes, NJ, USA) supplemented with 7% horse blood under the same microaerophilic conditions [7, 9]. *H. pylori* bacterial suspension was stored in Brucella broth (Difco, NJ, USA) with 10% dimethyl sulfoxide and 10% horse serum at − 80 °C.

### Antibiotic susceptibility test

We utilized the 2-fold agar dilution method to determine antibiotic susceptibility based on the EUCAST guideline [23]. We examined 5 antibiotics, including furazolidone (Tokyo Chemical Company, Tokyo, Japan) and rifabutin (Sigma Aldrich, St. Louis, MO, USA), which ranged from 0.063 to 8 μg/mL, and rifaximin (Tokyo Chemical Company), garenoxacin (Sigma Aldrich), and sitafloxacin (Haoyuan Chemexpress, Shanghai, China), which ranged from 0.063 to 32 μg/mL. Before performing the antibiotic susceptibility test, we subcultured *H. pylori* stored in Brucella broth twice on the Brucella agar plate supplemented with 7% horse blood for 3 days. After sufficient growth, we prepared Mueller–Hinton agar supplemented with 10% horse blood using a different concentration of antibiotics. Bacterial suspension collected from the agar plate was adjusted to an optical density of 0.1 and inoculated onto a blood agar plate containing a 2-fold serial dilution of antibiotics. The results were evaluated after incubating for 3–5 days in the microaerophilic environment. A strain was considered resistant if the minimum inhibitory concentration (MIC) exceeded the clinical breakpoints of > 4 mg/L for furazolidone and rifaximin and > 1 mg/L for rifabutin, garenoxacin, and sitafloxacin [20, 24–26]. We also determined the MIC50 from the median and the MIC90 from the 90th percentile measurement to represent the MIC of the antibiotic that is required to inhibit ≥50% and ≥ 90% of the strains, respectively.

### *H. pylori* mutation analysis

DNA was extracted from cultured *H. pylori* using Qiagen DNeasy Blood and Tissue Kit (Qiagen, Germany) following the manufacturer’s instructions. We used data from our previous studies for the *gyrA* and *gyrB* mutations from Nepalese strains [9]. To complete the data, we obtained *gyrA*, *gyrB,* and *rpoB* sequences from Bangladeshi strains from our next-generation sequencing (NGS) data (MiSeq; Illumina, Inc., San Diego, CA, USA) utilizing the BLAST algorithm implemented on CLC Genomic Workbench software (ver. 11; Qiagen, Venlo, the Netherlands). The queries for BLAST analysis were *hp0701*, *hp0501,* and *hp1198* from *H. pylori* 26,695 (GenBank accession number AE000511.1) for *gyrA*, *gyrB,* and *rpoB*, respectively. Briefly, after obtaining the sequence data, we confirmed that neither insertion nor deletion led to a frameshift mutation. Subsequently, we aligned the sequence based on the amino acid sequence using MAFFT (http://mafft.cbrc.jp/alignment/server/). For mutation related to rifaximin resistance, we compared all amino acids of resistant and sensitive strains to the reference sequence using our original PERL script and confirming by visual inspection. Variants identified in both the resistant and sensitive strains were considered normal and excluded from further analysis. Variants found in the resistant but not the sensitive strains might be related to antibiotic resistance. For mutation related to quinolone resistance, we compared the amino acids of all the resistant strains to strain 26,695 and searched to determine the presence of the variant at position 87 and 91 of *gyrA* and position 481, 483, and 484 of *gyrB* and the other mutations that were already published [9].

### Statistical analysis

Using multivariate analysis, we examined the effect of different variables on strain susceptibility and acquired odds ratios (OR) with 95% confidence intervals (CI). Variables assessed in the binary logistic regression analysis included sex, age, and clinical outcomes. A *P*-value < 0.05 was considered statistically significant. Spearman’s rank correlation coefficient was used to calculate the correlation between resistance rate and age. Susceptibility comparison between countries was also analyzed using the chi-squared test. IBM SPSS Statistics software version 23.0 (IBM Corp., Armonk, NY, USA) was used for all the statistical analyses.

## Results

### Antibiotic resistance rates

Table 1 summarizes the antibiotic resistance of *H. pylori* isolated in Nepal and Bangladesh including data for amoxicillin, metronidazole, and clarithromycin from our previous studies [7, 9].

**Table 1:** Comparison of antibiotic resistance rates in Nepal and Bangladesh

Antibiotic	Country (%)		
	Nepal (*n* = 42)	Bangladesh (*n* = 56)	Both countries (*n* = 98)
Current Study			
Garenoxacin^1^	12/42 (28.6)	29/56 (51.8)	41/98 (41.8)
Sitafloxacin	2/42 (4.8)	1/56 (1.8)	3/98 (3.1)
Furazolidone	0/42 (0.0)	0/56 (0.0)	0/98 (0.0)
Rifabutin	0/42 (0.0)	0/56 (0.0)	0/98 (0.0)
Rifaximin	22/42 (52.4)	36/56 (64.3)	58/98 (59.2)
Previous Study^2^			
Clarithromycin	9/42 (21.4)	22/56 (39.3)	31/98 (31.6)
Amoxicillin	0/42 (0.0)	2/56 (3.6)	2/98 (2.0)
Metronidazole	37/42 (88.1)	53/56 (94.6)	90/98 (91.8)
Tetracycline	0/42 (0.0)	0/56 (0.0)	0/98 (0.0)
Levofloxacin	18/42 (42.9)	37/56 (66.1)	55/98 (56.1)

^1^*P* = 0.041, garenoxacin resistance in Nepal vs. Bangladesh

^2^All data were obtained from our previous studies [7, 9]

The agar dilution method revealed that no strain was resistant to furazolidone and rifabutin, suggesting that these are a potentially effective regimen to eradicate clarithromycin- and levofloxacin-resistant *H. pylori* infection. We observed higher resistance to garenoxacin (51.8%) (29/56, 95% CI = 41.1–66.7%) in Bangladesh than in Nepal (28.6%) (12/42, 95% CI = 14.3–42.9%, *P* = 0.041). When combining these data with those from previous studies [7], we determined an association between garenoxacin and levofloxacin resistance in Nepal (*P* = 0.003). Although both sitafloxacin and garenoxacin were quinolones, sitafloxacin might be a prospective alternative regimen due to its low resistance rate (2/42, 4.8% in Nepal and 1/56, 1.8% in Bangladesh). Clarithromycin and sitafloxacin resistance was significantly correlated in Nepalese strains (*P* = 0.042). In Bangladesh, we identified a correlation between sitafloxacin and amoxicillin resistance (*P* = 0.036), although both of them had a low absolute number of resistant isolates. Rifaximin resistance in both countries was also high (64.3%, 36/56 vs. 52.4%, 22/42, *P* = 0.790).

Table 2 summarizes the multiple antibiotic resistance of *H. pylori* isolated in Nepal and Bangladesh.

**Table 2:** Single and Multiple Resistance Rates of *H. pylori* isolated from Nepal and Bangladesh

Antibiotics	Countries (%)		
	Nepal (*n* = 42)	Bangladesh (*n* = 56)	Both countries (*n* = 98)
True Single Resistance			
Metronidazole^a^	9/42 (21.4)	6/56 (10.7)	15/98 (15.3)
Levofloxacin^a^	0/42 (0.0)	1/56 (1.8)	1/98 (1.0)
Double Resistance^b^			
Levofloxacin+metronidazole	3/42 (7.1)	2/56 (3.6)	5/98 (5.1)
Levofloxacin+garenoxacin	0/42 (0.0)	1/56 (1.8)	1/98 (1.0)
Rifaximin+garenoxacin	2/42 (4.8)	0/56 (0.0)	1/98 (1.0)
Rifaximin+metronidazole	8/42 (19.0)	7/56 (12.5)	15/98 (15.3)
Rifaximin+clarithromycin	1/42 (2.4)	0/56 (0.0)	1/98 (1.0)
Clarithromycin+metronidazole	0/42 (0.0)	3/56 (5.4)	3/98 (3.0)
Triple Resistance			
Clarithromycin+levofloxacin+metronidazole	1/42 (2.4)	2/56 (3.6)	3/98 (3.1)
Clarithromycin+metronidazole+rifaximin	2/42 (4.8)	1/56 (1.8)	3/98 (3.1)
Levofloxacin+metronidazole+garenoxacin	2/42 (4.8)	3/56 (5.4)	5/98 (5.1)
Levofloxacin+metronidazole+rifaximin	3/42 (7.1)	4/56 (7.1)	7/98 (7.1)
Quadruple Resistance			
Clarithromycin+levofloxacin+metronidazole+rifaximin	0/42 (0.0)	1/56 (1.8)	1/98 (1.0)
Clarithromycin+levofloxacin+metronidazole +garenoxacin	2/42 (4.8)	2/56 (3.6)	4/98 (4.1)
Clarithromycin+metronidazole+rifaximin+garenoxacin	1/42 (2.4)	0/56 (0.0)	1/98 (1.0)
Levofloxacin+metronidazole+rifaximin+garenoxacin	4/42 (9.5)	8/56 (14.3)	12/98 (12.2)
Quintuple Resistance			
Clarithromycin+levofloxacin+metronidazole+garenoxacin+rifaximin	0/42 (0.0)	12/56 (21.4)	12/98 (12.2)
Amoxicillin+levofloxacin+metronidazole+garenoxacin+rifaximin	0/42 (0.0)	1/56 (1.8)	1/98 (1.0)
Sextuple Resistance			
Clarithromycin+levofloxacin+metronidazole+garenoxacin+rifaximin+sitafloxacin	2/42 (4.8)	0/56 (0.0)	2/98 (2.0)
Amoxicillin+levofloxacin+metronidazole+garenoxacin+rifaximin+sitafloxacin	0/42 (0.0)	1/56 (1.8)	1/98 (1.0)

^a^This number corresponds to our previous reports of the same strains, including clarithromycin, amoxicillin, metronidazole, tetracycline, and levofloxacin resistance

^b^Isolates categorized into a group will not be included in another group

Multiple resistance was defined as resistance to ≥2 antibiotics simultaneously [27] and consisted of double, triple, quadruple, quintuple, and sextuple resistance combined with clarithromycin, amoxicillin, metronidazole, levofloxacin, sitafloxacin, rifaximin, and garenoxacin. We exclusively categorized an isolate into either the multiple or single resistance group; therefore, no isolate was classified into 2 different groups. We observed higher resistance to multiple antibiotics in Bangladesh (87.5%) than in Nepal (78.6%). When we combined 10 antibiotics, we found that all antibiotics except for metronidazole (15.3%) and levofloxacin (1.0%) showed multiple resistance. Interestingly, we found an association between garenoxacin and rifaximin resistance in the Bangladeshi strains (*P* = 0.014), but not in the Nepalese strains (*P* = 0.320). We also found an association between garenoxacin and clarithromycin resistance, especially in Nepalese strains (*P* = 0.015). Meanwhile, rifaximin resistance was not related to clarithromycin and levofloxacin resistance, suggesting a specialized mechanism of resistance.

In Bangladesh, all garenoxacin-resistant strains were resistant to levofloxacin. All 3 sitafloxacin-resistant strains were also resistant to clarithromycin and occurred together with garenoxacin and rifaximin, further to metronidazole, clarithromycin, and levofloxacin. We also noted triple antibiotic resistance, with the highest prevalence in the combination of levofloxacin, metronidazole, and rifaximin in both countries (7/98, 7.1%) (Table 2). Among the Bangladesh population, quintuple resistance reached 23.2%, and the highest quadruple resistance was 14.3%.

### Garenoxacin and Rifaximin resistance correlated with sex and age

The distribution of resistance to garenoxacin and rifaximin between males and females was close to equal in both countries (*P* > 0.05, Additional file 1: Table S1). However, we saw a 6.4-fold increasing odds of rifaximin resistance in the group aged 40–49 years (95% CI = 1.156 to 35.437, *P* = 0.034, Additional file 1: Table S1) compared to the group aged > 59 years in the combined population. In addition, age and garenoxacin resistance had a significant negative correlation (*r* = − 0.207; 95% CI = − 0.418 to − 0.017, *P* = 0.041).

### Distribution of MIC

**Fig. 1:**
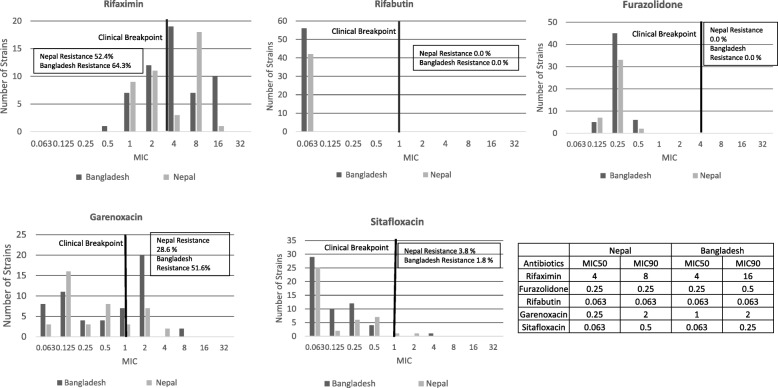
Minimal Inhibitory Concentration Distribution of Nepal and Bangladesh Strains The vertical line through the graph represents the resistance breakpoint. Although the MIC range from each antibiotic was diverse, a bimodal pattern was shown in rifaximin and garenoxacin, suggesting a primary resistance associated with the genetic mutation

The range of MIC from each antibiotic varied. Interestingly, a similar pattern of bimodal distribution was observed in the rifaximin and garenoxacin resistance rate in both countries (Fig. 1). The second peak of the bimodal pattern was spotted on the MIC ≤16 μg/mL, which may be associated with mutation related to low-level rifaximin resistance [28] and supported by the findings on mutation analysis. Rifabutin was very susceptible to all strains in both countries, exhibiting a very low MIC < 0.063 μg/mL. In furazolidone, most of the MIC was observed in 0.25 μg /mL (sensitive). Sitafloxacin MIC ranged from 0.063 to 4 μg/mL, but the majority was in the 0.063 μg/mL range (sensitive). However, a wide range of MIC (0.5 to 16 μg/mL) was observed in rifaximin and garenoxacin (0.063 to 8 μg/mL).

Among the rifamycin group, MIC90, which means the MIC of the antibiotic could eliminate 90% of the isolates, demonstrated that rifabutin was more effective than rifaximin was in the Bangladesh and Nepal strains, with 256 times lower MIC90 in rifabutin, suggesting a particular role of pharmacokinetic and pharmacodynamics in the human body. Furazolidone also demonstrated a relatively low MIC90 (0.25 μg/mL). Although we reported previously that Nepalese and Bangladeshi strains had high resistance to levofloxacin, our current results showed that MIC90 of garenoxacin was achieved at a concentration of 2 μg/mL, which was 8 times lower than for rifaximin. Sitafloxacin also demonstrated MIC90 4 times lower than for garenoxacin.

### Molecular detection

Rifamycins have a mechanism of action that inhibits bacterial RNA polymerase activity. Therefore, including rifaximin and rifabutin, resistance were related to mutation in the RNA polymerase gene or *rpoB* [29]. Table 3 shows the 10 highest numbers of locus mutation in *rpoB,* which was found in the rifaximin-resistant strains. Additional file 1: Table S2 summarizes the data. As shown in the previous study, which detected several mutations in *rpoB* associated with rifamycin [29], we found that isoleucine replaced valine amino acid in position 657 in only 3 strains (data are not shown). However, to our knowledge, no study has reported on a mutation related to rifaximin resistance. We confirmed that various point mutations, such as I2619V, appeared in 43.5% of resistance strains (Table 3**)**. Other mutations, such as V2592 L, T2537A, and F2538 L, might also be candidate mutations related to rifaximin but not rifabutin resistance. As reported, these mutations were located outside the known rifampin resistance-determining region [29], cluster I or cluster II [30], and were not related to high-level resistance (MIC ≥16 μg/mL).

**Table 3:** Mutation frequency of *rpoB*

No	Locus Mutation	Frequency (%)
1	I2619V	17 (43.6)
2	V2592 L	14 (35.9)
3	T2537A	14 (35.9)
4	F2538 L	14 (35.9)
5	K2359S	13 (33.3)
6	K2594R	13 (33.3)
7	D2381E	11 (28.2)
8	T1540A	7 (17.9)
9	N2603D	7 (17.9)
10	E2809D	7 (17.9)

Table 4 and Additional file 1: Table S3 shows the *gyrA* and *gyrB* mutations found in the Bangladeshi and Nepalese strains. We analyzed the presence of *gyrA* and *gyrB* mutations within fluoroquinolone drug-resistant strains. We used the data obtained from NGS to analyze the mutation in the Bangladeshi strains and our previously published data to analyze the mutation in the Nepalese strains [9].

**Table 4:** *gyrA* and *gyrB* mutations in garenoxacin and sitafloxacin resistance

*gyrA* mutation data					*gyrB* mutation data				
Mutation	Frequency	MIC levofloxacin	MIC garenoxacin	MIC sitafloxacin	Mutation	Frequency	MIC levofloxacin	MIC garenoxacin	MIC sitafloxacin
Bangladesh					Bangladesh				
N87K	9/29	4–16	1–2	0.063–0.5	D481E	1/29	2	2	0.25
D91N	8/29	2–64	1–8	0.063–0.25	R484K	1/29	64	8	4
D91G	5/29	2–4	1–2	0.063–0.25	D481E, R484K	14/29	2–64	1–8	0.063–0.5
D91Y	1/29	4	2	0.125	No mutation	13/29	4–16	1–2	0.063–0.5
N87K, D91N	1/29	64	64	2					
No mutation	5/29	4–16	1–2	0.063–0.25					
Nepal					Nepal				
N87K	3/11	32	1–2	0.5	E483K	1/10	32	4	0.5
N87I	1/11	32	4	0.5	No mutation	9/10	32	1–4	1–2
D91N	2/11	32	2	0.125–1					
D91Y	1/11	32	2	0.25					
N87K, D91N	1/11	32	4	2					
D91N, R130K	1/11	32	2	1					
D91N, S63P	1/11	0.125	2	0.5					
No mutation	1/11	32	1	0.5					

*MIC* Minimum inhibitory concentration, μg/mL

We observed a single mutation either in position 87 or 91 of the *gyrA* gene in the Nepalese and Bangladeshi garenoxacin-resistant strains (7/11 and 23/29, respectively), while several other strains did not possess any mutation (Table 4). In total, 1/11 strain from Nepal and 1/29 strain from Bangladesh had a double mutation at N87K and D91N and exhibited resistance to both garenoxacin and sitafloxacin with a considerably high MIC. Another sitafloxacin-resistant strain also had a double mutation at D91N and R130K. Thus, a mutation at position R130K might be a new candidate for sitafloxacin resistance.

As described previously, we evaluated the *gyrB* mutation, which consisted of a substitution in D481E and R484K (Table 4) [31]. Interestingly, most of the garenoxacin-resistant strains contained a double substitution in D481E and R484K (14/29; 48.3%).

## Discussion

We evaluated 5 antibiotics in an attempt to find an effective regimen to eradicate *H. pylori* infection in regions with high resistance to clarithromycin, metronidazole, and levofloxacin. We found that rifabutin and furazolidone were susceptible to all strains including those resistant to clarithromycin, levofloxacin, and metronidazole with a very low MIC. Our result agreed with several other studies also demonstrating a very low resistance rate in vitro and achieved a 96.6% cure rate in a clinical trial in several countries such as Japan, Italy, and Spain [26, 32, 33]. The effectiveness of rifabutin may be attributed to the basic physicochemical characteristics and increasing lipophilicity, tissue uptake, and intracellular concentration [34]. It was also absorbed better in the circulation and was a weak inducer of CYP450, thus, having better bioavailability [35]. Therefore, it becomes a recommended drug for rescue therapy in regions with high quinolone resistance rates as reported in the Maastricht V Consensus [8]. However, caution must be used due to its interaction with tuberculosis, which is prevalent in South Asia, contributing to 40% of the tuberculosis cases in the world [36], and cross-resistance to rifabutin and rifampicin was reported in several studies [37, 38]. Therefore, inappropriate use of rifabutin may affect tuberculosis treatment. In addition, several studies reported a cross-reaction with other drugs, and severe adverse effects, such as myelotoxicity, are a limitation of rifabutin, even though myelotoxicity can resolve on its own [39].

In addition, furazolidone showed promising results for both countries due to its zero resistance rate. The possible future application of this regimen in Nepal and Bangladesh was supported by several studies, which proposed furazolidone as the initial therapy regimen [40, 41], because it could achieve a > 90% cure rate in areas with high resistance to clarithromycin, metronidazole, or levofloxacin, such as China and Iran [42, 43]. The cost of furazolidone was also relatively lower compared to other drugs such as sitafloxacin and, thus, is more suitable in developing countries [44]. Even though furazolidone was reported to have a carcinogenic effect, the International Agency for Research on Cancer (IARC) categorized this regimen as Class 3, meaning “unclassifiable” regarding carcinogenicity in humans. This classification was less harmful than that for metronidazole, which was listed as Class 2B, signifying a “definite” carcinogen in animals and humans [22, 45, 46]. In addition, furazolidone-related side effects were reported to be relatively low [44].

Quinolone resistance is also a major problem in eradicating *H. pylori* not only in Nepal and Bangladesh but also worldwide. To gain a better understanding of this issue, we compared levofloxacin, garenoxacin, and sitafloxacin susceptibility and the mutation pattern in *gyrA* and *gyrB* from the same strains*.* Sitafloxacin demonstrated higher elimination capacity compared to garenoxacin and levofloxacin, which was supported by several previous studies from Japan proposing that sitafloxacin overcomes levofloxacin resistance, which was due to a mutation in *gyrA* [19, 20, 47]. Due to the potential therapeutic ability of sitafloxacin, further investigation with a larger sample size and its correlation with clinical outcome are necessary. However, garenoxacin susceptibility was low, which also supports our finding that garenoxacin resistance was related to levofloxacin. Although oral garenoxacin is safe without significant adverse effects [48], it may not suitable in Nepal and Bangladesh because the susceptibility was < 90% as recommended by the Maastricht V Consensus [8]. In contrast with the previous study reporting low resistance at a young age (< 30 years) [49], we found that resistance tended to increase in younger aged Nepalese individuals. Increased use of quinolones, such as levofloxacin, at an early age is related to poor susceptibility to garenoxacin [50], a quinolone frequently prescribed in children [51], multiple concurrent antibiotic prescriptions, and lack of education regarding usage were reported in Nepal [52]. The high prevalence of *Salmonella enterica* infection [53] increased the tendency for physicians to prescribe quinolones for gastroenteritis patients, even though it is actually virus related [51], and increased the quinolone resistance rate [54]. Tuberculosis is prevalent in South Asia, and a quinolone regimen, such as gatifloxacin, is also important for short-course treatment of multi-drug–resistant tuberculosis [55]. Thus, our results suggest that regulating the use of quinolones is necessary in South Asia.

We observed a bimodal pattern in the MIC distribution of rifaximin and garenoxacin, indicating a possible mechanism of acquired resistance, such as a genetic mutation [56]. In agreement with previous studies, molecular analysis for the *gyrA* and *gyrB* genes in the present study also showed that a single mutation in position 87 and 91 was related to a resistant phenotype in levofloxacin and garenoxacin [57], while double mutations (positioned at 87 and 91) were related to sitafloxacin resistance [16]. Interestingly, we also noted that sitafloxacin-resistant strains were all multi-drug resistant. Multiple resistance to quinolone drugs may be attributed to the change in membrane permeability [58], which may also play a role in multi-drug resistance shown by a decrease in drug accumulation inside the cells and alteration of the outer membrane protein (OMP) [59, 60]. Efflux genes present in *H. pylori* species such as efflux genes (*hefC)* [61] and resistance nodulation/division family (RND) [62]. The relationship of sitafloxacin to amoxicillin resistance may also cause multi-drug–resistance in sitafloxacin. Beta-lactam resistance due to alteration in *pbp IA* was reported to increase the MIC of quinolone, chloramphenicol, metronidazole, rifampin, and tetracycline [59]; however, further research is needed.

In the present study, we found that primary resistance to rifaximin was high in both countries, which coincides with the results of several previous studies [63, 64]. We should mention that the poor cure rate of rifaximin was probably due to its use as a single regimen; however, triple therapy of rifaximin together with a proton pump inhibitor and levofloxacin or clarithromycin also led to a poor cure rate in clinical studies, ranging from 30 to 58% [65]. Importantly, rifaximin had high safety ratings because it is poorly absorbed in the stomach, thus it barely approaches *H. pylori,* which lives under the gastric mucus and epithelium [66]. This safety feature may be useful for infection in children as described by a clinical trial in Russian children reporting an 85.4% cure rate; however, this may not be sufficient evidence due to the small number of subjects (41 patients), and the regimen included bismuth subcitrate and furazolidone [67]. We assessed the *rpoB* gene in rifaximin-resistant strains; however, only 3 strains had a mutation at V657I, as mentioned in previous studies [30, 63, 68]. We confirmed several novel mutations that appeared in almost half of the Nepalese and Bangladeshi strains, suggesting that another mutation or mechanism may have a higher influence than V657I. Further study should be performed to confirm how this mutation affects the enzymatic structure and resistance phenotype.

The spread of *H. pylori* strains containing resistance mutations may be related to garenoxacin and rifaximin resistance [69], especially in Bangladesh. A higher occurrence of multiple resistance was seen in the Bangladeshi compared to the Nepalese population, which is probably because Bangladeshi density was 10 times higher than in the Nepalese population, thus, facilitating the spread of resistant strains [7]. Mutation accumulation and acquisition of resistance plasmid may also play a role in quintuplet resistance, which occurs in sitafloxacin-resistant strains, thus, affecting the target side of another antibiotic [70].

The low number of participants in each country was the main limitation of the present study. Likewise, the MIC50 and MIC90 numbers in this study also should be interpreted with caution due to the small number of samples. We noted that, for *H. pylori,* there was no standardized clinical breakpoint of the antibiotics tested in this study by either EUCAST or CLSI, and the result may diverge from different methods. Mutation analysis was limited to the gene level, and strain relatedness was not established. Further investigation of the mutation and natural transformation may be necessary. Moreover, we provided the in vitro result only, which may need further confirmation from an in vivo study, as the true efficacy of the drug was affected by several elements such as pharmacodynamics and host factors. In spite of the limitations, this study has important findings and is probably the first to evaluate the susceptibility of salvage therapy in Nepal and Bangladesh, which is necessary for the future treatment of *H. pylori*. The limited availability of furazolidone and sitafloxacin in each country also deters the use of those antibiotics as primary therapy for *H. pylori.*

## Conclusions

We confirmed that all strains from Nepal and Bangladesh are susceptible to furazolidone and rifabutin. In addition, these countries have a very low rate of sitafloxacin-resistant strains, suggesting a possible future application for overcoming the resistance to clarithromycin, metronidazole, and levofloxacin. High resistance was observed in garenoxacin, which may be due to the presence of *gyrA* and *gyrB* mutations. Mutation analysis of *rpoB* could also explain the mechanism of rifaximin resistance. Therefore, these data could give a priori information to aid in choosing the more suitable regimen.

## Additional file

Additional file 1: **Table S1.** Distribution of antibiotic resistance in Nepal and Bangladesh patients. **Table S2.** Point Mutation in *gyrA* and *gyrB* in Bangladesh and Nepal. **Table S3.** Mutation in *rpoB* that was associated with rifaximin resistance. (DOCX 36 kb)

## Abbreviations

CI: Confidence interval
IARC: International Agency for Research on Cancer
MIC: Minimum inhibitory concentration
NGS: Next-generation sequencing
OR: Odds ratio

## References

[1] IARC Helicobacter pylori Working Group. Helicobacter pylori Eradication as a Strategy for Preventing Gastric Cancer. International Agency for Research on Cancer (IARC Working Group Reports, No 8) 2014. http://www.iarc.fr. Accessed 21 June 2018.

[2] Matsuhisa T, Aftab H (2012). Observation of gastric mucosa in Bangladesh, the country with the lowest incidence of gastric cancer, and Japan, the country with the highest incidence. Helicobacter.

[3] Ahmad MM, Ahmed DS, Rowshon AH, Dhar SC, Rahman M, Hasan M (2007). Long-term re-infection rate after helicobacter pylori eradication in Bangladeshi adults. Digestion.

[4] Vilaichone RK, Mahachai V, Shiota S, Uchida T, Ratanachu-ek T, Tshering L (2013). Extremely high prevalence of helicobacter pylori infection in Bhutan. World J Gastroenterol.

[5] Yamaoka Y (2018). How to eliminate gastric cancer-related death worldwide?. Nat Rev Clin Oncol.

[6] Ford AC, Forman D, Hunt RH, Yuan Y, Moayyedi P (2014). Helicobacter pylori eradication therapy to prevent gastric cancer in healthy asymptomatic infected individuals: systematic review and meta-analysis of randomised controlled trials. BMJ.

[7] Aftab H, Miftahussurur M, Subsomwong P, Ahmed F, Khan AK, Yamaoka Y (2016). Helicobacter pylori antibiotic susceptibility patterns in Bangladesh: emerging levofloxacin resistance. J Infect Dev Ctries.

[8] Malfertheiner P, Megraud F, O'Morain CA, Gisbert JP (2017). Management of Helicobacter pylori infection-the Maastricht V/Florence Consensus Report. Gut.

[9] Miftahussurur M, Shrestha PK, Subsomwong P, Sharma RP, Yamaoka Y (2016). Emerging helicobacter pylori levofloxacin resistance and novel genetic mutation in Nepal. BMC Microbiol.

[10] Thirumurthi S, Graham DY (2012). Helicobacter pylori infection in India from a western perspective. Indian J Med Res.

[11] Graham DY, Fischbach L (2010). Helicobacter pylori treatment in the era of increasing antibiotic resistance. Gut.

[12] Hunt R, Xiao S, Megraud F, Leon-Barua R, Bazzoli F, Van der Merwe S (2011). Helicobacter pylori in developing countries. World gastroenterology organisation global guideline. J Gastrointestin Liver Dis.

[13] Kunin CM (1996). Antimicrobial activity of rifabutin. Clin Infect Dis.

[14] Brogden RN, Fitton A (1994). Rifabutin. A review of its antimicrobial activity, pharmacokinetic properties and therapeutic efficacy. Drugs.

[15] Akada JK, Shirai M, Fujii K, Okita K, Nakazawa T (1999). In vitro anti-helicobacter pylori activities of new Rifamycin derivatives, KRM-1648 and KRM-1657. Antimicrob Agents Chemother.

[16] Mori H, Suzuki H, Matsuzaki J, Masaoka T, Kanai T (2017). Antibiotic resistance and gyrA mutation affect the efficacy of 10-day sitafloxacin-metronidazole-esomeprazole therapy for helicobacter pylori in penicillin allergic patients. United European Gastroenterol J.

[17] Scarpignato C, Pelosini I (2005). Rifaximin, a poorly absorbed antibiotic: pharmacology and clinical potential. Chemotherapy.

[18] Fung-Tomc JC, Minassian B, Kolek B, Huczko E, Aleksunes L, Stickle T (2000). Antibacterial spectrum of a novel des-fluoro(6) quinolone, BMS-284756. Antimicrob Agents Chemother.

[19] Suzuki H, Nishizawa T, Muraoka H, Hibi T (2009). Sitafloxacin and Garenoxacin may overcome the antibiotic resistance of helicobacter pylori with gyrA mutation. Antimicrob Agents Chemother.

[20] Murakami K, Okimoto T, Kodama M, Tanahashi J, Fujioka T, Ikeda F (2009). Sitafloxacin activity against helicobacter pylori isolates, including those with gyrA mutations. Antimicrob Agents Chemother.

[21] Alba C, Blanco A, Alarcon T (2017). Antibiotic resistance in helicobacter pylori. Curr Opin Infect Dis.

[22] Graham DY, Lu H (2012). Furazolidone in helicobacter pylori therapy: misunderstood and often unfairly maligned drug told in a story of French bread. Saudi J Gastroenterol.

[23] Eucast D (2000). Document EDEF. 3.1, June 2000: determination of minimum inhibitory concentrations (MICs) of antibacterial agents by agar dilution. Clin Microbiol Infect.

[24] Ogata SK, Gales AC, Kawakami E (2014). Antimicrobial susceptibility testing for helicobacter pylori isolates from Brazilian children and adolescents: comparing agar dilution, E-test, and disk diffusion. Braz J Microbiology.

[25] Adachi JA, DuPont HL (2006). Rifaximin: a novel nonabsorbed rifamycin for gastrointestinal disorders. Clin Infect Dis.

[26] Nishizawa T, Suzuki H, Matsuzaki J, Muraoka H, Tsugawa H, Hirata K (2011). Helicobacter pylori resistance to Rifabutin in the last 7 years. Antimicrob Agents Chemother.

[27] Zhang Y-X, Zhou L-Y, Song Z-Q, Zhang J-Z, He L-H, Ding Y (2015). Primary antibiotic resistance of helicobacter pylori strains isolated from patients with dyspeptic symptoms in Beijing: a prospective serial study. World J Gastroenterol.

[28] Dec M, Wernicki A, Puchalski A, Urban-Chmiel R (2015). Antibiotic susceptibility of lactobacillus strains isolated from domestic geese. Br Poult Sci.

[29] Hays C, Burucoa C, Lehours P, Tran CT, Leleu A, Raymond J. Molecular characterization of Helicobacter pylori resistance to rifamycins. Helicobacter. 2018;23(1):e12451. 10.1111/hel.12451 29168600

[30] Heep M, Odenbreit S, Beck D, Decker J, Prohaska E, Rieger U (2000). Mutations at four distinct regions of the rpoB gene can reduce the susceptibility of helicobacter pylori to rifamycins. Antimicrob Agents Chemother.

[31] Hu Y, Zhang M, Lu B, Dai J (2016). Helicobacter pylori and antibiotic resistance, a continuing and intractable problem. Helicobacter.

[32] D'Elios MM, Silvestri E, Emmi G, Barnini T, Prisco D (2012). Helicobacter pylori: usefulness of an empirical fourth-line rifabutin-based regimen. Expert Review Gastroenterol Hepatol.

[33] Ciccaglione AF, Tavani R, Grossi L, Cellini L, Manzoli L, Marzio L (2016). Rifabutin containing triple therapy and Rifabutin with bismuth containing quadruple therapy for third-line treatment of helicobacter pylori infection: two pilot studies. Helicobacter.

[34] Jabès D, Della Bruna C, Rossi R, Olliaro P (1994). Effectiveness of rifabutin alone or in combination with isoniazid in preventive therapy of mouse tuberculosis. Antimicrob Agents Chemother.

[35] Sousa M, Pozniak A, Boffito M (2008). Pharmacokinetics and pharmacodynamics of drug interactions involving rifampicin, rifabutin and antimalarial drugs. J Antimicrob Chemother.

[36] Basnyat B, Caws M, Udwadia Z (2018). Tuberculosis in South Asia: a tide in the affairs of men. Multidiscip Respir Med.

[37] Tan Y, Hu Z, Zhao Y, Cai X, Luo C, Zou C (2012). The beginning of the rpoB gene in addition to the rifampin resistance determination region might be needed for identifying rifampin/rifabutin cross-resistance in multidrug-resistant mycobacterium tuberculosis isolates from southern China. J Clin Microbiol.

[38] Berrada ZL, Lin S-YG, Rodwell TC, Nguyen D, Schecter GF, Pham L (2016). Rifabutin and rifampin resistance levels and associated rpoB mutations in clinical isolates of mycobacterium tuberculosis complex. Diag Microbiol Infect Dis.

[39] Gisbert JP, Calvet X (2012). Review article: rifabutin in the treatment of refractory helicobacter pylori infection. Aliment Pharmacol Ther.

[40] Hajaghamohammadi A, Safiabadi Tali SH, Samimi R, Oveisi S, Kazemifar AM (2014). Low dose furazolidone for eradication of H- pylori instead of clarithromycin: a clinical trial. Glob J Health Sci.

[41] Xie Y, Zhang Z, Hong J, Liu W, Lu H, Du Y (2018). Furazolidone-containing triple and quadruple eradication therapy for initial treatment for Helicobacter pylori infection: A multicenter randomized controlled trial in China. Helicobacter.

[42] Xie Y, Zhu Y, Zhou H, Lu ZF, Yang Z, Shu X (2014). Furazolidone-based triple and quadruple eradication therapy for helicobacter pylori infection. World J Gastroenterol.

[43] Mohammadi M, Attaran B, Malekzadeh R, Graham DY (2017). Furazolidone, an underutilized drug for H. Pylori eradication: lessons from Iran. Dig Dis Sci.

[44] Zhuge L, Wang Y, Wu S, Zhao RL, Li Z, Xie Y (2018). Furazolidone treatment for helicobacter pylori infection: a systematic review and meta-analysis. Helicobacter.

[45] Auro A, Sumano H, Ocampo L, Barragán A (2003). Evaluation of the carcinogenic effects of furazolidone and its metabolites in two fish species. Pharmacogenomics J.

[46] Cancer IAfRo (1982). Some food additives, feed additives and naturally occurring substances.

[47] Sugimoto M, Sahara S, Ichikawa H, Kagami T, Uotani T, Furuta T (2015). High helicobacter pylori cure rate with sitafloxacin-based triple therapy. Aliment Pharmacol Ther.

[48] Gajjar DA, Bello A, Ge Z, Christopher L, Grasela DM (2003). Multiple-dose safety and pharmacokinetics of oral garenoxacin in healthy subjects. Antimicrob Agents Chemother.

[49] Ji Z, Han F, Meng F, Tu M, Yang N, Zhang J (2016). The Association of age and Antibiotic Resistance of helicobacter pylori: a study in Jiaxing City, Zhejiang Province. China Medicine.

[50] Rose L, Coulter MM, Chan S, Hossain J, Di Pentima MC (2014). The quest for the best metric of antibiotic use and its correlation with the emergence of fluoroquinolone resistance in children. Ped Infect Dis J.

[51] Shankar PR, Upadhyay DK, Mishra P, Subish P, Dubey AK, Saha AC (2007). Fluoroquinolone utilization among inpatients in a teaching hospital in western Nepal. J Pak Med Assoc.

[52] Basnyat B, Pokharel P, Dixit S, Giri S (2015). Antibiotic use, its resistance in Nepal and recommendations for action: a situation analysis. J Nepal Health Res Counc.

[53] Crump JA, Sjölund-Karlsson M, Gordon MA, Parry CM (2015). Epidemiology, clinical presentation, laboratory diagnosis, antimicrobial resistance, and antimicrobial management of invasive Salmonella infections. Clin Microbiol Rev.

[54] Bhetwal A, Maharjan A, Khanal PR, Parajuli NP (2017). Enteric fever caused by Salmonella enterica Serovars with reduced susceptibility of fluoroquinolones at a community based teaching Hospital of Nepal. Int J Microbiol.

[55] Sotgiu G, Tiberi S, Centis R, D’Ambrosio L, Fuentes Z, Zumla A (2017). Applicability of the shorter ‘Bangladesh regimen’ in high multidrug-resistant tuberculosis settings. Int J Infect Dis.

[56] Ammor MS, Flórez AB, Van Hoek AH, Clara G, Aarts HJ, Margolles A (2008). Molecular characterization of intrinsic and acquired antibiotic resistance in lactic acid bacteria and bifidobacteria. J Mol Microbiol Biotechnol.

[57] Rimbara E, Noguchi N, Kawai T, Sasatsu M (2012). Fluoroquinolone resistance in helicobacter pylori: role of mutations at position 87 and 91 of GyrA on the level of resistance and identification of a resistance conferring mutation in GyrB. Helicobacter.

[58] Hooper DC, Jacoby GA (2015). Mechanisms of drug resistance: quinolone resistance. Ann N Y Acad Sci.

[59] Kwon DH, Dore M, Kim J, Kato M, Lee M, Wu J (2003). High-level β-lactam resistance associated with acquired multidrug resistance in helicobacter pylori. Antimicrob Agents Chemother.

[60] Liu Z-Q, Zheng P-Y, Yang P-C (2008). Efflux pump gene hefA of helicobacter pylori plays an important role in multidrug resistance. World J Gastroenterol.

[61] Kutschke A, de Jonge BLM (2005). Compound efflux in <em>helicobacter pylori</em>. Antimicrob Agents Chemother.

[62] Saidijam M, Benedetti G, Ren Q, Xu Z, Hoyle CJ, Palmer SL (2006). Microbial drug efflux proteins of the major facilitator superfamily. Curr Drug Targets.

[63] Holton J, Vaira D, Menegatti M, Barbara L (1995). The susceptibility of helicobacter pylori to the rifamycin, rifaximin. J Antimicrob Chemother.

[64] Gerard L, Garey KW, DuPont HL (2005). Rifaximin: a nonabsorbable rifamycin antibiotic for use in nonsystemic gastrointestinal infections. Expert Rev Anti-Infect Ther.

[65] De Giorgio R, Stanghellini V, Barbara G, Guerrini S, Ferrieri A, Corinaldesi R (1997). Rifaximin and helicobacter pylori eradication. Eur Rev Med Pharmacol Sci.

[66] Gasbarrini A, Gasbarrini G, Pelosini I, Scarpignato C (2006). Eradication of helicobacter pylori: are rifaximin-based regimens effective?. Digestion.

[67] Nizhevich AA, Shcherbakov PL, Akhmadeeva EN, Khasanov R (2011). Rifaximin in combined treatment of the helicobacter pylori infection in childhood. Exp Clin Gastroenterol.

[68] Heep M, Beck D, Bayerdörffer E, Lehn N (1999). Rifampin and Rifabutin resistance mechanism in helicobacter pylori. Antimicrob Agents Chemother.

[69] Adamek RJ, Suerbaum S, Pfaffenbach B, Opferkuch W (1998). Primary and acquired helicobacter pylori resistance to clarithromycin, metronidazole, and amoxicillin--influence on treatment outcome. Am J Gastroenterol.

[70] Jacoby GA (2005). Mechanisms of resistance to quinolones. Clin Infect Dis.

