# Supplementary material for: Effective therapeutic regimens in two South Asian countries with high resistance to major Helicobacter pylori antibiotics
Source: Antimicrob Resist Infect Control. 2019 Feb 15;8:40. doi: 10.1186/s13756-019-0482-x (PMC6377755; doi:10.1186/s13756-019-0482-x)
Supplement: Supplementary file 1 — Table S1. Distribution of antibiotic resistance in Nepal and Bangladesh patients. Table S2. Point Mutation in gyrA and gyrB in Bangladesh and Nepal. Table S3. Mutation in rpoB that was associated with rifaximin resistance. (DOCX 36 kb) [file 13756_2019_482_MOESM1_ESM.docx]

Suppl. Table 1. Distribution of antibiotic resistance in Nepal and Bangladesh patients

| Characteristic | n | Resistant regiment (%) | | |
| --- | --- | --- | --- | --- |
|  |  | Rifaximin | Garenoxacin | Sitafloxacin |
| **Both Countries** | |  |  |  |
| Sex (%) |  |  |  |  |
| Male | 42 | 24/42 (57.1) | 20/42 (47.6) | 0/42 (0.0) |
| Female | 56 | 34/56 (60.7) | 21/56 (37.5) | 3/56 (5.4) |
|  |  |  |  |  |
| Age (%) |  |  |  |  |
| <30 | 27 | 15/27 (55.6) | 15/27 (55.5) | 0/27 (0.0) |
| 30-39 | 26 | 15/26 (57.7) | 12/26 (46.1) | 1/26 (3.8) |
| 40-49 | 21 | 16/21 (76.2) | 7/21 (33.3) | 1/21 (4.8) |
| 50-59 | 15 | 9/15 (60.0) | 4/15 (26.7) | 1/15 (6.7) |
| >59 | 9 | 9/15 (60.0) | 4/15 (26.7) | 0/15 (0.0) |
|  |  |  |  |  |
| Clinical Outcome (%) | | |  |  |
| Gastritis | 88 | 53/88 (60.22) | 40/88(45.5) | 3/88(3.5) |
| Duodenal Ulcer | 4 | 2/4(50) | 0/4(0.0) | 0/4(0.0) |
| Peptic Ulcer | 3 | 2/3 (66.7) | 1/3(33.3) | 0/3(0.0) |
| Gastric cancer | 3 | 1/3(33.3) | 0/3(0.0) | 0/3(0.0) |
|  |  |  |  |  |
| **Nepal** |  |  |  |  |
| Sex (%) |  |  |  |  |
| Male | 16 | 8/16 (50.0) | 5/16 (31.3) | 0/16 (0.0) |
| Female | 26 | 14/26 (53.8) | 7/26 (26.9) | 2/26 (7.7) |
|  |  |  |  |  |
| Age (%) |  |  |  |  |
| <30 | 10 | 4/10 (40.0) | 4/10 (40.0) | 0/10 (0.0) |
| 30-39 | 7 | 5/7 (71.4)* | 2/7 (28.6) | 0/7 (0.0) |
| 40-49 | 11 | 8/11 (72.7)* | 3/11 (27.3) | 1/11 (9.1) |
| 50-59 | 7 | 4/7 (57.1) | 2/7 (28.6) | 1/7 (14.3) |
| >59 | 7 | 1/7 (14.3) | 1/7 (14.3) | 0/7 (0.0) |
|  |  |  |  |  |
| Clinical Outcome (%) | |  |  |  |
| Gastritis | 35 | 19/35(54.3) | 12/25(34.3) | 2/35(5.7) |
| Duodenal Ulcer | 4 | 2/4(50.0) | 0/4 (0.0) | 0/4 (0.0) |
| Gastric cancer | 3 | 1/3(33.3) | 0/3(0.0) | 0/3(0.0) |
|  |  |  |  |  |
| **Bangladesh** |  |  |  |  |
| Sex (%) |  |  |  |  |
| Male | 26 | 16/26 (61.5) | 15/26 (57.7) | 0/26 (0.0) |
| Female | 30 | 20/30 (66.7) | 14/30 (46.7) | 1/30 (3.3) |
|  |  |  |  |  |
| Age (%) |  |  |  |  |
| <30 | 17 | 11/17 (64.7) | 11/17 (64.7) | 0/17 (0.0) |
| 30-39 | 19 | 10/19 (52.6) | 10/19 (52.6) | 1/19 (5.3) |
| 40-49 | 10 | 8/10 (80.0) | 4/10 (40.0) | 0/10 (0.0) |
| 50-59 | 8 | 5/8 (62.5) | 2/8 (25.0) | 0/8 (0.0) |
| >59 | 2 | 2/2 (100) | 2/2 (100) | 0/2 (0.0) |
|  |  |  |  |  |
| Clinical Outcome (%) | |  |  |  |
| Gastritis | 53 | 34/53 (64.2) | 28/53 (52.8) | 1/53 (1.9) |
| PUD | 3 | 2/3 (66.7) | 1/3 (33.3) | 0/3 (0.0) |

*P = 0.03, OR=16, CI 95% 1.315-194.623

**P = 0.047, OR=15, CI 95%= 1.030-218.300

PUD: peptic ulcer disease

Suppl. Table 2. Point Mutation in *gyrA* and *gyrB* in Bangladesh and Nepal

| Country | Strain | Levofloxacin MIC (µg/mL) | | Garenoxacin MIC (µg/mL) | | Sitafloxacin MIC (µg/mL) | | *gyrA* Mutation | *gyrB* Mutation |
| --- | --- | --- | --- | --- | --- | --- | --- | --- | --- |
| Bangladesh | BGD003 | R | 4 | R | 2 | S | 0.5 | N87K | None |
| Bangladesh | BGD010 | R | 4 | S | 0.5 | S | 0.25 | N87K | None |
| Bangladesh | BGD013 | R | 2 | R | 2 | S | 0.25 | D91N | D481E, R484K |
| Bangladesh | BGD027 | R | 4 | R | 2 | S | 0.125 | D91N | D481E, R484K |
| Bangladesh | BGD034 | R | 2 | R | 2 | S | 0.25 | D91N | D481E |
| Bangladesh | BGD038 | R | 2 | R | 2 | S | <0.063 | D91G | D481E, R484K |
| Bangladesh | BGD043 | R | 4 | R | 2 | S | 0.25 | D91N | None |
| Bangladesh | BGD049 | R | 8 | R | 2 | S | 0.25 | N87K | None |
| Bangladesh | BGD052 | R | 8 | R | 1 | S | 0.125 | N87K | D481E, R484K |
| Bangladesh | BGD054 | R | 4 | S | 0.125 | S | <0.063 | None | D481E, R484K |
| Bangladesh | BGD063 | R | 64 | R | 8 | R | 4 | N87Y, D91N | R484K |
| Bangladesh | BGD065 | R | 4 | S | 0.25 | S | <0.063 | None | D481E, R484K |
| Bangladesh | BGD069 | R | 8 | R | 2 | S | 0.25 | N87K | D481E, R484K |
| Bangladesh | BGD070 | R | 8 | S | 0.5 | S | 0.125 | N87K | None |
| Bangladesh | BGD072 | R | 8 | R | 1 | S | 0.25 | None | D481E, R484K |
| Bangladesh | BGD073 | R | 8 | R | 1 | S | 0.25 | N87K | None |
| Bangladesh | BGD076 | R | 4 | R | 2 | S | 0.125 | None | None |
| Bangladesh | BGD077 | R | 2 | S | 0.125 | S | <0.063 | D91N | R484K |
| Bangladesh | BGD078 | R | 4 | S | 0.5 | S | <0.063 | D91N | None |
| Bangladesh | BGD081 | R | 4 | R | 1 | S | 0.125 | D91N | D481E, R484K |
| Bangladesh | BGD082 | R | 8 | S | 0.5 | S | 0.125 | N87K | D481E, R484K |
| Bangladesh | BGD084 | R | 16 | R | 2 | S | 0.25 | N87K | None |
| Bangladesh | BGD086 | R | 16 | R | 2 | S | 0.5 | N87K | None |
| Bangladesh | BGD090 | R | 4 | R | 2 | S | 0.25 | D91G | D481E, R484K |
| Bangladesh | BGD091 | R | 4 | R | 2 | S | 0.125 | D91Y | None |
| Bangladesh | BGD097 | R | 64 | R | 8 | S | 0.5 | D91N | D481E, R484K |
| Bangladesh | BGD101 | R | 4 | R | 2 | S | 0.125 | D91G | None |
| Bangladesh | BGD103 | R | 16 | R | 2 | S | 0.25 | None | None |
| Bangladesh | BGD104 | R | 8 | R | 2 | S | 0.25 | D91N | None |
| Bangladesh | BGD107 | R | 16 | R | 1 | S | <0.063 | N87K | D481E, R484K |
| Bangladesh | BGD109 | R | 4 | R | 2 | S | <0.063 | None | None |
| Bangladesh | BGD111 | R | 4 | S | 0.25 | S | <0.063 | N87K | D481E, R484K |
| Bangladesh | BGD112 | R | 2 | R | 2 | S | <0.063 | D91G | D481E, R484K |
| Bangladesh | BGD114 | R | 2 | R | 2 | S | <0.063 | D91N | D481E, R484K |
| Bangladesh | BGD120 | R | 8 | R | 2 | S | 0.5 | N87K | D481E, R484K |
| Bangladesh | BGD130 | R | 4 | R | 1 | S | 0.125 | D91G | D481E, R484K |
| Bangladesh | BGD133 | R | 4 | R | 1 | S | 0.125 | None | None |
| Nepal | NP2 | R | >32 | S | 0.5 | S | 0.5 | N87K | None |
| Nepal | NP5 | R | >32 | S | 0.5 | S | 0.25 | D91G | None |
| Nepal | NP8 | R | >32 | R | 2 | S | 0.25 | D91N | None |
| Nepal | NP16 | R | >32 | S | 0.5 | S | 0.063 | S63P, D91N | None |
| Nepal | NP18 | R | >32 | S | 0.5 | S | 0.063 | D91N | None |
| Nepal | NP24 | S | 0.125 | R | 2 | S | 0.5 | D91N, S63P | None |
| Nepal | NP29 | R | >32 | R | 2 | S | 0.5 | S63P, N87K, P188S | None |
| Nepal | NP38 | R | >32 | S | 0.5 | S | 0.25 | D99V | None |
| Nepal | NP49 | R | >32 | R | 4 | R | 2 | N87K, D91N, V172I | None |
| Nepal | NP55 | R | >32 | R | 4 | S | 0.5 | N87I | E483K |
| Nepal | NP70 | R | >32 | S | 0.125 | S | 0.063 | None | None |
| Nepal | NP86 | R | >32 | R | 1 | S | 0.5 | N87K | None |
| Nepal | NP89 | R | >32 | R | 2 | R | 1 | D91N, R130K | None |
| Nepal | NP90 | R | >32 | R | 2 | S | 0.5 | N87K | None |
| Nepal | NP120 | R | 8 | S | 0.5 | S | 0.25 | A88P | None |
| Nepal | NP123 | R | >32 | R | 2 | S | 0.25 | D91Y | None |
| Nepal | NP140 | R | >32 | R | 2 | S | 0.125 | D91N | None |
| Nepal | NP141 | R | >32 | S | 0.5 | S | 0.125 | D91N | None |
| Nepal | NP142 | R | >32 | R | 1 | S | 0.5 | S63P, R130K | None |

Suppl. Table 3. Mutation in *rpoB* that was associated with rifaximin resistance

| **Strain name** | **MIC (**µg/mL) | **Mutation list** |
| --- | --- | --- |
| Bangladesh 3 | 16 | T635A, E970Q, T1540A, E2809D |
| Bangladesh 10 | 8 | R1173H, E1529D, S1702N, I2497V, E2809D |
| Bangladesh 13 | 4 | K231E, N1599S, D2381E, V2592L, K2532N, T2537A, F2538L, K2359S, I2619V, E2809D |
| Bangladesh 16 | 4 | R812C, S906N, T1540A, E1573Q, G2242R, R2314H, S2376G, D2381E, V2592L, T2537A, F2538L, K2359S, R2608C, I2619V, V2803M |
| Bangladesh 24 | 16 | R812C, V1126I, D2381E |
| Bangladesh 27 | 4 | I516T, S988L, I1076T, V1567I, N2000H, R2007K, R2208H, A2358V, R2365C, D2450N, A2473T, I2497V |
| Bangladesh 34 | 16 | D108N, I325T, A851V, S2416G, V2592L, K2532N, T2537A, F2538L, K2359S, I2619V |
| Bangladesh 38 | 4 | T137I, I994V, R1173C, A1367V, D1942N, T2333M, D2381E, K2419Q, D2520G, I2619V |
| Bangladesh 42 | 4 | - |
| Bangladesh 43 | 16 | E46Q, T635A, V1595A, A1644V, T2076S, A2415V, E2809D |
| Bangladesh 46 | 4 | A353V, A1118F, E1573Q, K1768R, E2212K, E2315K, S2376R, V2592L, T2537A, F2538L, K2359S, I2619V |
| Bangladesh 49 | 4 | V1053A, M1144I, T1540A, N2603D, R2642K |
| Bangladesh 52 | 8 | M252I, R812C, H1058Y, T1165I, P1218H, T1540A, H1770Y, S1908N, V2592L, T2537A, F2538L, K2359S, P2550L, I2687V, V2803M, M2874I |
| Bangladesh 53 | 4 | M86I, K416R, P1188S, D1983G, N2000H, V2036I, K2703E |
| Bangladesh 54 | 4 | A267T, T635I, A1574V, P1965T, D2381E, R2642K |
| Bangladesh 63 | 16 | K270R, A874T, M1239I, T1540A, N1599S, T2247I, D2381E, V2592L, T2537A, F2538L, K2359S, K2594R, I2619V, S2623V |
| Bangladesh 69 | 8 | E62G, T315A, E755K, T842I, T886A, E997G, T1540A, V1614A, R2314H, V2592L, T2537A, F2538L, K2359S, I2619V |
| Bangladesh 70 | 8 | V657I, S1198F, K1211N, M1239I, L1784I, A1948S, I2619V, R2776H, E2814K, D2887N |
| Bangladesh 72 | 4 | V811M, L1784F, R2365H, V2592L, T2537A, F2538L, K2359S, I2619V, T2844I |
| Bangladesh 73 | 4 | T635A, E2809D |
| Bangladesh 76 | 4 | E1185K, A1382V, N2603D |
| Bangladesh 82 | 4 | N773S, V779I, A874T, V1387M, G2234D, D2381E |
| Bangladesh 84 | 16 | M291R, V956A, I1505T, D2381E, N2603D, E2685G |
| Bangladesh 90 | 4 | S165F, A2235T, P2277L, K2422R, K2738R |
| Bangladesh 91 | 8 | A890T, V1682M, R2365H, V2592L, K2532N, T2537A, F2538L, K2359S, I2619V |
| Bangladesh 94 | 4 | V24I. T137I, A790T, A954V, Y1554H, N1599S, D2381E, V2592L, T2537A, F2538L, K2359S, I2619V |
| Bangladesh 97 | 16 | R101H, N1599S, D2381E, I2V |
| Bangladesh 107 | 4 | N236D, A890V, V1595A, I2222V, A2256V, A2358V, V2592L, T2537A, F2538L, K2359S, I2619V |
| Bangladesh 109 | 16 | A473V, V657I, T1586A, V2036I, R2642K |
| Bangladesh 110 | 4 | D610N, V657I, A2455V, R2478H, I2619V, R2776H |
| Bangladesh 112 | 8 | E961G, D1577G, D2178G, T2219M, R2365H, V2592L, T2537A, F2538L, K2359N, I2619V |
| Bangladesh 114 | 8 | A1382V, A1890V, D2450N, R2642K |
| Bangladesh 119 | 16 | S662N, A1118T, N1599S, D2081N, V2317I, N2603D |
| Bangladesh 120 | 16 | V779I, A874T, H1001Y, A1026S, L1026S, D1163N, T1540A, S2376R, D2381E, K2419Q, A2455V, D2499N, V2592L, T2537A, F2538L, K2359S, I2619V |
| Bangladesh 127 | 4 | T1540A, A1574V, L1784I, P1965T, A2455V |
| NP24 | 8 | R101H, V2214M, A2455V, I2619V |
| NP110 | 8 | K488E, P1636S, K2419Q, V2592L, T2537A, F2538L, K2359S, I2619V |
| NP114 | 8 | S879I, G2380S, A2415V, S2623V |
| NP145 | 8 | N26D, L310I, V779I, A1018T, K2072R, T2076S, S2623V, L2882I |
